# The rise of plant-based milk alternatives: exploring nutritional, health, and sustainability impacts

**DOI:** 10.1016/j.fochx.2026.103528

**Published:** 2026-01-14

**Authors:** Noor Asif, Oneeza Anwar, Sabika Arif, Zahra Anwar, Iahtisham-Ul-Haq, Sezai Ercisli, Robert Mugabi, Gulzar Ahmad Nayik

**Affiliations:** a Kauser Abdulla Malik School of Life Sciences, Forman Christian College (A Chartered University), Lahore, Punjab, Pakistan; b Department of Horticulture, Faculty of Agriculture, Ataturk University, 25240 Erzurum, Türkiye; c HGF Agro, ATA Teknokent, Erzurum, Türkiye; d Department of Food Technology and Nutrition, Makerere University, Kampala, Uganda; e Marwadi University Research Centre, Department of Microbiology, Marwadi University, Rajkot 360003, Gujarat, India

**Keywords:** Plant-based milk alternatives, Allergens, Health effects, Nutritional benefits, Consumer trends, Food standards

## Abstract

The rising demand for plant-based milk alternatives (PBMAs) reflects growing consumer interest in sustainable nutrition and healthier diets. This review provides a comprehensive examination of the nutritional quality, health impacts, and sustainability of PBMAs derived from cereals, legumes, nuts, and seeds. It analyzes their physicochemical characteristics, fortification strategies, and advancement in processing technologies, including high-pressure homogenization, ultrasonication, and enzymatic treatments, which enhance shelf life, sensory quality, and bioavailability. This review also explores allergenicity and antinutritional factors associated with PBMAs, highlighting the role of formulation and processing in addressing these limitations. In addition, it discusses global market trends, consumer perceptions, and regulatory considerations, underscoring the role of PBMAs in shaping sustainable and resilient food systems. By addressing knowledge gaps across nutrition, processing, and sustainability, this review provides valuable insights for researchers, industry professionals, and policymakers seeking to optimize PBMAs for a growing, health-conscious, and eco-aware global population.

## Highlights

• Explores nutrition and sustainability of plant-based milk alternatives.
• Evaluates processing methods for quality and fortification of PBMAs.
• Examines allergenicity and antinutritional factors in plant-based milks.
• Analyzes global market trends and consumer perceptions of PBMAs.
• Provides insights for creating nutritious, sustainable plant-based milks.

## 1 Introduction

As awareness of the relationship between diet, health, and sustainability increases, noticeable shifts in global dietary patterns have been observed in recent years. Greater access to scientific information through digital media has contributed to heightened consumer interest in foods perceived as healthier, environmentally sustainable, and ethically produced. One prominent outcome of this shift is the growing demand for PBMAs, which are liquid products derived from cereals, legumes, nuts, and seeds and formulated to serve as substitutes for dairy milk. The rising popularity of PBMAs is driven by multiple factors, including lactose intolerance, milk protein allergies, vegan and flexitarian dietary preferences, and concerns regarding the environmental footprint of animal-based food systems (Moss et al., 2022).

PBMAs are designed to replicate selected sensory, functional, and physicochemical attributes of dairy milk, such as appearance, mouthfeel, and emulsified structure, enabling their use in beverages, cooking, and food formulations (McClements, Newman, & McClements, 2019; Sethi, Tyagi, & Anurag, 2016). Advances in processing technologies and fortification strategies have further enhanced their nutritional profile and consumer acceptability, allowing these products to function as practical dietary alternatives rather than conventional beverages. Although PBMAs differ fundamentally from dairy milk in origin and composition, they are widely recognized by consumers as non-dairy substitutes that can be used in similar culinary contexts. Accordingly, this review consistently refers to these products as PBMAs, ensuring clarity, regulatory consistency, and accurate scientific communication.

The popularity of plant-based dairy alternatives is rising, with sales expected to surge in the upcoming years. The compound annual growth rate (CAGR) of the plant-based milk market from 2018 to 2022 was 7.9 % and is anticipated to exhibit a CAGR of 9.9 % for 2023–2033 (*Plant-Based Milk Market Insights 2025–2035, 2023*). According to Fortune Business Insight, the global market of plant-based dairy alternatives was 28.55 billion USD in 2023 and is expected to reach 32.38 billion USD by 2025 (*Dairy Alternatives Market Size, Share, Growth Report, 2032*, 2024).

PBMAs are substitutes for dairy products sourced from animals that are formed from the homogenization of plant parts (either fruit or seed) with water to mimic the taste and consistency of conventional milk (Silva & Smetana, 2022)*.* As a greater number of people are following vegetarian and vegan diets, the demand for diverse plant-based dairy alternatives has also skyrocketed. Plant-based milk products are the most sought-after product among all types of plant-based dairy alternatives because the extraction of plant material is relatively simple, has high consumer acceptance, and is available in the marketing settings (Research, 2025). Cashew and Almond milk are the most desired and accepted plant-based milks due to their mild and sweet taste and absence of coarse appearance (Reyes-Jurado et al., 2023).

Many consumers tend to choose plant-based dairy alternatives to avoid health concerns linked with the use of dairy sources (Moss et al., 2022). The nutritional profile of the final product depends on the type of plant chosen and treatment methods. Such as when soybeans were fermented for a short time 28.5 % increase in the total phenolics was observed; similarly, when soybeans were subjected to fermentation for 72 h, a 78 % increase was seen. Improving the constituents through different techniques can help treat health threats such as diabetes, hypercholesterolemia, hypertension, cancer, etc. (Reyes-Jurado et al., 2023). Generally, plant-based dairy alternatives are unable to meet the daily requirements for calcium, vitamin B12, and vitamin B6. The protein content of average plant-based dairy alternatives is significantly lower than that of cow's milk, but the dietary fiber content is greater in oat milk as compared to that of cow's milk (Pointke et al., 2022a, 2022b). As soy milk has 8.7 mg/100 mL of protein and cow milk has 3.28 mg/100 mL of protein, PBMAs don't need to meet all the nutritional aspects (Reyes-Jurado et al., 2023). Many consumers have turned to plant-based dairy alternatives due to milk allergy and lactose intolerance. Similarly, many people consume plant-based dairy alternatives to avoid health implications associated with dairy and dairy products, like high serum cholesterol levels and high caloric content. Some other health concerns from cow milk are milk protein allergy, often found in infants and children, lactose intolerance, mainly in the old age population, and antibiotic residues (Antunes et al., 2023; Reyes-Jurado et al., 2023). Depending on the type of product consumed, plant-based dairy alternatives generally provide 8–9 % less saturated fat than low-fat dairy milk (Craig et al., 2023).

Climate change and environmental disturbances are also contributing to an environmentally conscious dietary pattern. People concerned about health, sustainable agricultural practices, and ethical production of food are more likely to consume plant-based dairy alternatives (Boaitey & Minegishi, 2020). The media has also played an essential part in shaping the perceptions of consumers, as a plant-based diet is often advertised as environmentally friendly. This is also true for plant-based dairy alternatives, as vegan diets produce 50 % less greenhouse gases than modern omnivorous diets (Scarborough et al., 2023).

A comprehensive review of the published literature regarding PBMAs was conducted. The inclusion criteria included articles published in peer-reviewed journals from 2010 to 2024. Articles that were not peer-reviewed or were in a language other than English were excluded. The websites of regulatory bodies, including FAO, USDA, CAC, and EFSA, were also consulted for relevant regulatory and nutritional guidelines. The databases that were primarily searched were Google Scholar, PubMed, Scopus, and ScienceDirect. Specific keywords were used to search these databases, such as ‘Plant-based milk’, ‘Plant-based milk alternatives’, ‘Plant-based substitutes’, or specific ingredients like ‘almond milk’, ‘oat milk’, etc.

The available literature on plant-based milk shows a lack of comprehensive data regarding various plant-based milk and milk products. Most articles about plant-based milk focus on the nutritional composition and environmental impact of plant-based milk and provide a comparison between dairy milk and PBMAs. However, most literature lacks a thorough discussion of the impact of processing methods on the nutritional profile of plant-based milk and an in-depth analysis of water usage, carbon footprint, and greenhouse gas emissions associated with plant-based milk.

The objective of this study is to provide a comprehensive analysis of plant-based dairy alternatives, including their macronutrient and micronutrient profiles, the presence of allergens, market trends, environmental impact, including water usage, and life cycle assessment. This study also aims to examine the labeling regulations and quality standards of PBMAs, along with consumer behavior towards plant-based milk, as well as recent developments and barriers faced by the alternative dairy industry.

## 2 Types and processing techniques of plant-based dairy alternatives

**Fig. 1:**
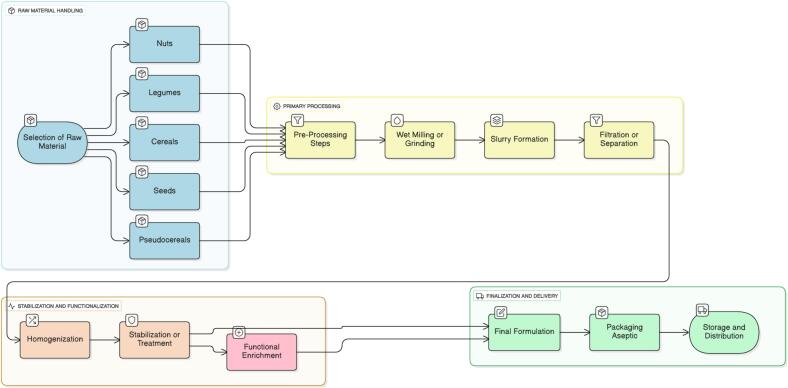
Processing flow of plant-based milk alternatives showing key steps from raw material preparation to packaging, with functional enrichment using natural sweeteners, arabinoxylans, and antioxidant bioactives to improve nutrition and functional.

PBMAs are colloidal suspensions or emulsions, which consist of dissolved and disintegrated plant material”. They are water-based extracts made from legumes, cereals, pseudo cereals, oilseeds, vegetables, and nuts. The word milk is labeled as beverages, drinks, and dairy alternatives (Reyes-Jurado et al., 2023). The value of PBMAs is increasing daily as more people switch from dairy-based diets to plant-based ones, considering them healthy, eco-friendly, and cruelty-free. Processing plant milk involves several steps from the selection of raw material to the packaging of the finished product. All the steps must be followed according to the provided directions so that the customers can receive appealing, wholesome, nutritious, and safe products. The classification based on categories is given in Table 1. Several methods to produce milk from different plant sources, and the procedure for manufacturing these alternatives are described in the flow chart below, Fig. 1. A few convenient plant milk processing techniques are available that benefit the manufacturers (Daryani, Pegua, & Aryaa, 2024). So, the processing of some common plant milks is given as follows.

**Table 1:** Classification of Plant Based Milk Alternatives based on categories.

Category	Sources
Cereal Based	Corn milk, Spelt milk, Rice milk, Oat milk
Legume Based	Soy milk, Peanut milk, Lupin milk, Cowpea milk
Nut Based	Almond milk, Coconut milk, Hazelnut milk, Pistachio milk, Walnut milk
Seed Based	Sesame milk, Flax milk, Hemp milk, Sunflower milk
Pseudo Based	Quinoa milk, Teff milk, Amaranth milk

### 2.1 Hemp milk

Hemp milk is extracted from the homogenization of ground hemp (*Cannabis sativus)* seeds (Beşir, Awad, & Mortaş, 2022)*.* The hemp seeds naturally have little to no amounts of tetrahydrocannabinol (THC), but during harvesting, they can be exposed to other parts, such as flowers, and get contaminated. Exposure to THC can cause serious health concerns such as gastrointestinal issues, sedation, and psychological events (Arango et al., 2024; Beşir et al., 2022).

Hemp seeds cultivated to have less than 0.3 % tetrahydrocannabinol (THC) alkaloid are mixed with hot water, with a mixing ratio of five parts of water to one part of hemp seeds. The hemp seeds and water are heated at 180–200 °F until a slurry is formed, which is circulated through a wet mill for loop grinding. A partially ground, milky mixture is obtained, which is recirculated until the standard values for Brix levels, total solids percentage, and pH are obtained. The recirculated slurry is then filtered to remove the shells, fibers, and other large solids. The filtered hemp slurry with 16 % solids is homogenized, and the white, creamy hemp milk is collected in a receiving tank. The hemp base is quickly cooled down to a temperature of less than 50 °F to prevent oxidation of oils and vitamins; otherwise, rancidity may occur. This hemp base can be customized with the addition of stabilizers, emulsifiers, and sweeteners (Ramos-Sanchez et al., 2025).

Hemp milk is an emulsion of oil and water and has a high tendency to coalesce, flocculate, and generate cream, thus making it extremely unstable with a short shelf life. Natural emulsifiers extracted from hemp protein have been used, but they showed low emulsifying capacity. Some enzymatic treatments can be used, which enhance the emulsifying capacity of hemp protein, which will eventually increase the stability of the milk (Thakur & Kasankala, 2025). Research conducted by Naylor (2021) manufactured hemp seed milk by adding sunflower lecithin as an emulsifying agent in 3 %, 5 %, and 7 % quantities. 3 % and 5 % treatments showed a 15-day shelf life, whereas 7 % showed the longest shelf life. Synthetic emulsifiers can be added to improve the stability of hemp milk. However, it increases the cost of production and is also associated with certain inflammatory health conditions. Homogenization techniques such as High-pressure Homogenization (HPH) can be used to reduce the rancidity of hemp milk. Homogenization pressure provides energy that disrupts the oil droplets, causing the proteins to form new interfaces with the oil surface. In addition, ultrasonic treatment is a cost-effective method that optimizes polyphenols, improving the shelf-life of hemp milk without causing adverse health impacts (Paul, Kumar, Kumar, & Sharma, 2020).

The shelf-life of hemp milk is correlated with the concentration of hemp, with the highest concentration (7 % hemp milk) showing the longest shelf-life (Naylor, 2021). Hemp milk has low heat stability as its protein tends to denature and aggregate, so heat treatments must maintain a temperature of less than 80 °C (Beşir et al., 2022).

Aseptic processing can extend the shelf-life of hemp milk to almost a year while ensuring a chalk-white color and good flavor. This process involves sterilization by exposing the hemp milk to ultra-high temperatures (195 °F–285 °F) for 3–15 s and cooling the milk rapidly. The hemp milk is then packed under sterile packaging conditions. This process ensures the least thermal stress while maximizing the safety of the product (Thakur & Kasankala, 2025).

### 2.2 Almond milk

Almond milk is a thick, creamy, milky white drink similar to cow milk in color and consistency (Reyes-Jurado et al., 2023). In many studies, it is found that almonds are first roasted (mostly at 95–100 °C for 30 min) for the manufacturing of its milk which reduces the concentrations of benzaldehyde and pyrazine to 0.05 ppm, so that, its natural taste can be obtained (Romulo, 2022). After that, the almonds are peeled either by using water (soaking overnight or an 18–20 h period), acid, or base (Aydar, Tutuncu, & Ozcelik, 2020a, 2020b). After that, blanching is done, which helps to reduce the microbes and inactivate the enzymes present in almonds. The almonds undergo a wet milling process, which involves adding water and then grinding them, followed by filtration. The addition of different ingredients is done to improve the quality of the almond milk. Sterilization is done after that to extend the shelf life of milk, and then homogenization is done so that its stability can be increased, and mostly the homogenization of almond milk is done at 350 MPa (UHP) and 85 °C (Romulo, 2022). In the end, almond milk is packed and stored at 4 °C.

There are many methods by which almond milk can be obtained, and its characteristics can be improved. If ultrasonication is used at optimized conditions to produce almond milk, a reduction in the amounts of *E coli* and *Listeria monocytogenes* can be achieved. Similarly, increasing the processing time leads to a decline in viscosity and suspended particle size that ensures physical stability and an improved Brix level (Sharma, Gayathri, & Priya, 2018). The use of a pulsed ultrasound technique at 20 kHz, while increasing the processing time (1, 4, 8, 12, and 16 min), can improve the in vitro digestibility of almond milk protein (Vanga, Wang, Orsat, & Raghavan, 2020). Also, high-pressure homogenization (HPH) can be used to get almond milk, and the best stability of almond milk by this process was obtained at 172 MPa at 85 °C for 30 min in a high-pressure homogenizer (Sharma et al., 2018).

### 2.3 Soy milk

Soy milk and almond milk share similar characteristics in terms of flavor and consistency. They appear white in color and thick in consistency (De, Shrivastav, Das, & Goswami, 2022). Soy milk formation mostly starts from soaking soy in water, and it is done to achieve its softness, which reduces the time of blanching (Saini & Morya, 2021). After that, wet milling is done by grinding soybeans using boiling water. After that filtration is done so that the milk part and the cake part of the raw material can be separated. This can be done with a muslin cloth, filter paper, or double-layered cheesecloth. After that, fortification or enrichment of milk is done if needed. One of the solutions to increase the protein content is to add or use a material that has high protein content, and whose sensory profile matches soy milk, for that different lentils can be used. When enrichment or fortification is done, soy milk is sterilized so that its shelf life can be extended, and homogenization is done so that its clarity, stability, and whiteness index can be increased. In the end, aseptic packaging is done to increase the shelf life and stored in a cool and dry place. The shelf life of soybean milk is 90 days at ambient temperature and 170 days at refrigeration temperature.

There are also some innovative techniques to get soy milk with improved qualities. High-pressure processing is one of the techniques that can improve the characteristics of soy milk (Rajan et al., 2023). Pulse electric field is an option to get soy milk with improved characteristics as it causes a reduction in fatty acids and helps in conserving more compounds than the other treatments, as heat processing causes many compound losses and reduces bacterial load (Hariono, Brilliantina, Kautsar, Wijaya, & Kurnianto, 2024). UV radiation is also used to deactivate *Salmonella enterica* and make milk safer. Martínez-García et al. (2023) found that continuous short-wave ultraviolet (UV-C) treatment using a tubular annular thin film reactor led to a drastic reduction in microbial counts in soy milk, effectively inactivating *Listeria monocytogenes, Escherichia coli,* and spores of *Bacillus subtilis* and *Aspergillus niger*.

### 2.4 Oat milk

It is not milk but a water extract of oats with a smooth milk-like flavor (Yu et al., 2023). It is a type of cereal-based milk that looks and feels like regular dairy milk (Yu et al., 2023). Canada has the highest production of oats. *Avena sativa* L. and *Avena nuda* L. are the two most popular species of oats used globally for oat milk production (Cui, Jia, Zhao, Hou, & Zhou, 2023). From 2018 to 2019, an increase in sales of more than USD 60 million has been reported for oat milk. The very first brand of oat milk was created in 1990 by a Swedish scientist. The purpose was to reduce environmental impact and meet specific dietary needs. Water extracted from oats looks and feels more like milk and is called oat milk. That is why it is not a true milk derived from an animal source. It shares huge market shares and serves as a sufficient source for both macronutrients and micronutrients, along with dietary fibers (Yu et al., 2023). The low lipid content, high amount of unsaturated fatty acids, and antioxidant properties make oat milk a healthy alternative to animal-based milk (Zhou et al., 2023). Oat milk also has advantages in providing protection against diseases like cancer and preventing high blood glucose and cholesterol levels. The nutrient profile of oat milk is the same as oats, except that some of it is lost during the manufacturing process. Therefore, the nutrient level needs to be adjusted by developing more efficient processing methods or fermentation techniques. As per results, the sensory profile of oat milk among several other PBMAs is considered to be the closest one to dairy milk (Yu et al., 2023). Processing of oat milk involves soaking for 8 h, which enhances the taste (Cui et al., 2023), grinding of oat tissues, enzymatic hydrolysis to obtain oat slurry, separation, filtration, heat treatment, and homogenization (Yu et al., 2023). Enzymatic hydrolysis and an increase in temperature enhance the fluid properties (Cui et al., 2023). Type of processing has a reducing effect on macronutrient content and quality of oat milk. Factors like temperature determine the fluid properties. Fat globule preparation is done with emulsifiers, additives, and oil separated from plant sources, which are then mixed with water. Water that is used must be treated either by heating, reverse osmosis, or filtration. Different oils like coconut oil, olive oil, sunflower oil, and corn oil are used for emulsification. Oat milk needs fortification with minerals, vitamins, and calcium carbonate to meet the nutrient profile of dairy-sourced milk. The presence of micronutrients enables microorganisms to feed on them, causing spoilage. Research has proven that the use of ultra-high-pressure homogenization (UHPH) can increase the shelf life from 3 to 57 days. Ultrasonication has proven a reduction in the growth of *Listeria monocytogenes* and *Escherichia coli* O157:H7, hence resulting in improved quality and increased shelf life as well (Yu et al., 2023).

### 2.5 Coconut milk

Coconut milk, which is produced by using mature coconuts, is easy to digest and acts as an instant source of energy due to the presence of medium-chain triglycerides. This is an attribute unique to coconut milk among all the other PBMAs. According to some research, coconut milk has a fat known as lauric acid, which can help fight cancer, improve the immune system, and support brain development. It is used globally in several bakery products. It's a rich source of vitamin E, which has anti-aging properties with rare allergic reactions. Adding more to the list of advantages, coconut milk consumption aids in digestion, has a cooling effect, enhances skin health, and acts as a source of micronutrients like calcium, potassium, magnesium, and zinc (Tulashie, Amenakpor, Atisey, Odai, & Akpari, 2022).

Coconut in raw form can come shelled for processing. If unshelled, then dehulling is done, which is the removal of the outer shell by soaking the coconut in hot water. Then, blanching or steam cooking is done, as both have a thermal effect, and they will eventually reduce the microbial load and enzymatic activity. After blanching, the next step is wet milling, which is the addition of water at 80 °C for 10 min, followed by grinding. The water quantity, temperature, and feed in wet milling determine the standard of the final product. Filtration is performed to separate the milk and the cake. The ingredients are added to increase stability and prevent oxidation. Fortification and enrichment are performed to increase protein, minerals, and vitamins. Sterilization is done to increase shelf life and maintain quality. Microfiltration can be used as an alternative, which is a no-heat sterilization procedure that removes microorganisms and increases the shelf life as well. Homogenization is done to achieve stability, white color, and clarity in the milk. To further increase shelf life and stability, aseptic packaging and cold storage at 4 °C are done (Aydar et al., 2020a, 2020b).

### 2.6 Challenges in the processing of plant-based milk

A vital step in the processing of the plant-based milk is the heat treatment. Various thermal processes, such as pasteurization, ultra-heat treatment (UHT), and sterilization, can be utilized. These thermal processes are mainly employed due to their ability to reduce microbial growth. These treatments must be carefully selected and should be optimized based on the type of plant-based milk being processed (Romulo, 2022).

Overheating can degrade the amino acids and trigger deteriorative reactions that can negatively impact the nutritional composition of the final product (Popova & Mihaylova, 2019; Silva, Silva, & Ribeiro, 2020). High hydrostatic pressure (HHP) and high-pressure homogenization (HPH) can be used as alternative methods. These techniques use high pressure (100-600 MPa) and low temperatures (30–85 °C) to improve the shelf-life and stability of products without compromising their texture or nutritional quality (Romulo, 2022). The heat processes also denature and aggregate protein present in soy-based milk, affecting the solubility of proteins and the quality of the final product. A non-thermal process called pulsed electric field (PEF) can be used as an alternative. PEF enhanced the rheological properties of soy-based milk, in addition to minimizing the negative effects of thermal treatment (Sethi et al., 2016).

Oats are a rich source of starch (55–60 %), which is gelatinized when heated in the presence of water, producing a gel-like consistency and reducing the fluidity of oat-based milk. Before heat treatment, the starch must be hydrolyzed to prevent its gelatinization (Silva et al., 2020). Many manufacturers are adding micronutrients to improve the nutritional composition of plant-based milk products. However, these nutrients tend to degrade in the presence of heat or oxygen (Silva et al., 2020). The metal ions present in certain micronutrients can react with other nutritional components, and sequestrants may be added to maintain the stability of the product (Zhang et al., 2024).

A small shelf-life is another challenge faced during the processing of PBMAs. PPBMAs are colloidal solutions made from large-sized dispersed particles such as starch granules or fat globules (Sethi et al., 2016). The sedimentation of these particles can result in a chalky or sandy product with very low storage life (Silva et al., 2020). To overcome this issue, the size of the solid particles can be reduced through homogenization. Homogenization breaks down aggregates and lipid droplets, preventing the coalescence of fat particles, improving the shelf-life of the final product (Silva et al., 2020).

## 3 Organoleptic properties

The two main concerns of PBMA are bad aroma and poor taste (Vaikma, Kaleda, Rosend, & Rosenvald, 2021). Milk substitutes give a gritty texture, so to overcome it, thickeners and sugars are added to please the customers (Sunidhi, Vij, & Katoch, 2021). Natural sweeteners such as monk fruit extract, stevia, and fruit-derived syrups contribute sweetness with minimal caloric load while also offering secondary antioxidant benefits. Their extraction, purification, and current applications in the food industry highlight their suitability for PBMAs, particularly in formulations aimed at reducing added sugar levels while improving sensory acceptance.

Structural polysaccharides such as arabinoxylans are increasingly used to modify the rheology and mouthfeel of plant-based milks. Their ability to bind water, increase viscosity, enhance foam stability, and act as soluble dietary fiber makes them attractive as clean-label structuring agents. Comprehensive reviews of arabinoxylan recovery and functionality indicate that they can be incorporated during grinding, slurry preparation, or even post-homogenization, depending on the desired final texture. These fibers also support the stability of fortified PBMAs enriched with phenolic compounds, antioxidants, or nutraceuticals by improving matrix integrity and reducing phase separation. (Castro-Muñoz et al., 2022). PBMAs often give off odors because of protein hydrolysis or lipid oxidation, but cow milk only gives off a certain flavor. PBMAs typically have their unique aroma profiles, such as nutty, beany, cooked grain, green, starchy, or caramel, which depend on the protein source used (Grossmann, Kinchla, Nolden, & McClements, 2021). Sensory hedonic analysis was done to check the sensory characteristics of the four types of milk (hemp, oats, quinoa, and almond). Based on color, almond milk was liked the most of all, and the least liked was oat milk; the remaining two fell in between. Based on odor, from best to worst, were: quinoa, hemp, almond, and oats. On overall taste and liking, almond milk was liked the most, and quinoa milk was least liked (Jemaa, Gamra, Falleh, Ksourı, & Bejı, 2021). The PBMAs with the least similar taste to cow's milk were least preferred by consumers. The better the taste, the better the sales (Giacalone, Clausen, & Jaeger, 2022).

Nut-based alternatives give a nutty flavor, which is why consumers often don't prefer them. As it is a cow milk alternative in the market, they expect a similar taste and flavor to cow's milk. The off flavors are overcome by adding vanilla and cocoa or by forming blends with alternative sources (Giacalone et al., 2022). Chickpea milk is yellowish and darker than cow's milk in color and tastes like beans, as they belong to the class of legumes. The presence of isoflavones and saponins gives the beany flavor. Coconut milk has an appearance like cow's milk; it gives a whitish color because of the white color of the coconut. A blend was formed using chickpeas and coconut to enhance all the characteristics in terms of nutrition, flavor, color, aroma, and texture (Rincon, Botelho, & de Alencar, 2020).

The beany and earthy flavor of the milk made from legume-based milk is caused by the lipid oxidation of the compounds (n-hexanal and n-hexanol). The aftertaste is also sometimes caused by bioactive compounds such as isoflavonoids. The color scale ranges from whites, greens, and browns, depending on the base material. The insoluble particles of the legumes give off a chalky and sandy texture in the mouth (Irondi et al., 2025). The milk made from lentils showed a similar viscosity to the cow's milk (Grossmann et al., 2021). Hydrophilic polymers such as guar gum or locust bean gum were added to plant milk to improve its viscosity and enhance the mouthfeel as well (Huang et al., 2024).

Soy milk possesses a beany flavor, and almond milk gives a nutty flavor with a sweet note. The natural pigments in oats and soybeans give a brownish color to the plant milks (McClements, 2020). Due to the presence of large insoluble particles, soy milk has a chalky and grainy texture (Giacalone et al., 2022). Cashew milk gives a creamy, viscous solution. Oat milk has a gentle, sweet impression with a creamy consistency that makes it a good substitute for cow's milk. Hemp milk has a strong, earthy, nutty flavor and thick, creamy consistency, best for savory dishes. Coconut milk gives a mild coconut-like flavor (Grossmann et al., 2021).

A survey was conducted by Moss et al. (2022) of plant-based milk by 323 Canadians. The parameters on which the alternatives were assessed were creaminess, thickness, and graininess. The evaluation was done using the hedonic scale and CATA (Check All That Apply). In this study, no comparison with cow's milk was made, and the basis of evaluation was between plant milks only. The milk substitutes used for the evaluation were oat, soy, cashew, chickpea, coconut, and almond milk. The results showed that people preferred pea and almond milk over others. The consumers preferred rich and thick consistency milk over the ones with watery consistency. Peanut milk was preferred more than soy milk.

The study evaluated 9 samples of plant milk available in the Estonian market. Cereal and pseudo-cereal-based milk were 42 %, nut-based milk 41 %, legume milk 16 %, and seed-based milk constituted 1 %. RATA (Rate All That Apply) and volatile compound analysis (GC–MS) were used for sensory evaluation. Cereal-based milk had a bitter flavor, while buckwheat and quinoa samples had a sweet taste. The consistency of these cereal milks was like water. Rice milk gave a sharp taste with an aroma of hay. “Besides nuttiness, almond beverages may taste salty, soapy, may have a sweet or roasty odor, and possess a thicker, lumpier texture.” Coconut milk and almond milk had similar flavor and consistency. The category of nut milk resembled the taste of cow's milk. “In addition to leguminous taste and odor, soy beverages were often characterized by metallic and astringent taste, hay-like and earthy odor, and red-tinted in their appearance.” The seed category contained only hemp seed beverages, which gave it a hay-like odor (Vaikma et al., 2021).

Fermentation helps in increasing the organoleptic properties of the plant-based milks. Use of various probiotic bacteria reduces the off-flavor of PBMAs and increases the desirable dairy-like aroma in them (Engels et al., 2022). It is reported in the research that when soy-based beverages are fermented with *Lactobacillus harbinensis* M1, it produces 2,3-butanedione and acetone, which improve the sensory attributes, including the buttery aroma (Zheng et al., 2020). The same is reported with the hemp seed milk, that when it is fermented by probiotic bacteria, certain chemicals are produced which produce a buttery flavor and a pleasant mouthfeel. (Peng et al., 2023). There are also many cereal-based drinks that, when fermented with different *lactobacillus* strains, exhibited pleasant sensory attributes (Moiseenko, Glazunova, & Fedorova, 2024). An increase in the whiteness index along with luminosity has also been observed in almond milk when its fermentation was done by the mixed culture of *S. thermophilus* and *Lactobacillus reuteri* (Bernat, Cháfer, Chiralt, & González-Martínez, 2015). So, fermentation helps in increasing the organoleptic properties and ovrall acceptance of PBMAs, thereby improving the customers' willingness to consume them. Fermentation of various food wastes has also resulted in the successful production of valuable by-products, including enzymes, pigments, and biofuels (Siddiqui et al., 2023).

As a substitute, consumers expect it to be like cow's milk in terms of flavor, color, texture, mouthfeel, etc. The most preferred milk substitute is oat milk because of its color and sweet flavor, like cow's milk. The more resemblance to the cow's milk, the more the purchases.

## 4 Nutritional content

PBMAs have their pros and cons, where they overcome the issue of allergenicity and health concerns, but also lack mammalian milk characteristics such as cholesterol and saturated fatty acids. They provide higher amounts of various vitamins, minerals, and fiber than cow's milk (Daryani et al., 2024). PBMA has lower amounts of protein than cow milk. They have proteins with low digestibility compared to animal proteins. It either lacks or has no vitamins, especially vitamin D and B_12_ (Antunes, Bexiga, Pinto, Roseiro, & Quaresma, 2022). It can cause a deficiency if only alternative milk is taken in the diet. The blends of two or more alternatives can influence the nutritive value of milk (Silva & Smetana, 2022). The blend of chickpea and coconut milk provided more nutritional quantities in terms of proteins, lipids, minerals, and vitamins than cow's milk (Rincon et al., 2020). “Based on the nutrient and sensory profile, it can be implied that soy almond milk blend suits well as a candidate for use as a non-dairy milk alternative” (Sunidhi et al., 2021). Some anti-nutritional compounds, such as oxalates and phytates, reduce the bioavailability as well.

Preprocessing treatments such as roasting, dehulling, blanching, sprouting, etc., are done to reduce the amounts of anti-nutrients and enhance the mouthfeel of these milks (Daryani et al., 2024). Processing impacts the nutrient content as some water-soluble vitamins leach out or are lost during processing, such as in soaking or bleaching, resulting in low amounts of vitamins (Munekata et al., 2020). The thermal process also destroys heat-sensitive nutrients and reduces protein digestibility and amino acid availability. To overcome this issue, supplementation is done, and some alternate processing technology is introduced, such as High-Pressure Homogenization (HPH), High Hydrostatic Pressure (HHP), Pulse Electric Field (PEF), and Ultrasound (Aydar et al., 2020a, 2020b). Application of acid, heat, and enzymatic actions coagulates the seeds' protein. Cow milk protein is flexible and forms a gel-like structure in yogurt and cheeses. Whereas, plant proteins are compact, forming different structures; hence, due to the absence of disordered protein, they give a rough texture, unlike cow's milk, which gives a smooth and delicate texture (McClements, 2020).

Another easy and affordable method used to produce milk alternatives is fermentation, which does not require blending and increases the nutritional and sensorial properties. It also increases the shelf life concerning microbial activity. Fermentation can increase the content of protein in PBMAs by growing the microbes, which are food-grade, along with improving the solubility of proteins present in plants. As an example, fermentation of soybeans with *Lactobacillus plantarum* increases the beneficial amino acids like _l-_Lysine, and fermentation with *Bifidobacterium* increases the overall crude protein content of the soy-based milks and other drinks (Song, Frías, Martínez-Villaluenga, Vidal-Valdeverde, & De Mejia, 2008). Fermentation in rice milk helps to break down the anti-nutritional factors with the help of lactic acid bacteria, which also helps to enhance the iron, magnesium, and calcium content. This aids in the immunity of organs and digestion by increasing the beneficial bacteria (Sharma et al., 2018).

PBMAs are fortified with minerals to overcome deficiencies, especially calcium, which is added to meet daily requirements (El Sadig & Wu, 2024). Calcium and vitamin D are fortified in PBMAs, but the assurance of their bioavailability is still unknown (Rincon et al., 2020). Vitamin A, D, and calcium are fortified in plain milk during manufacturing (Sunidhi et al., 2021).

The most widely used legume for milk alternatives is soybeans. Soymilk provides a good profile of proteins considering all essential amino acids, a considerate amount of both soluble and insoluble dietary fibers (35 %), a good ratio of Polyunsaturated Fatty Acids to Saturated Fatty Acids (82:18), and a good mineral profile comprising of calcium, potassium, iron, magnesium, zinc, and copper (Olías, Delgado-Andrade, Padial, Marín-Manzano, & Clemente, 2023). Soy is a leading substitute for cow's milk, and people showing allergic reactions to cow's milk are likely to show allergenicity towards soy milk as well (El Sadig & Wu, 2024). Soy milk is the nearest replicate to cow's milk in terms of calcium and complete protein content. 105 cal, 4 g of fats, and 6 g of protein are present in 8 oz of soy milk (Sunidhi et al., 2021). Chickpeas have a good protein proportion with a range of 20.9 to 25.27 % (Pachekrepapol, Kokhuenkhan, & Ongsawat, 2021).

Out of the nut category, almonds are used mostly in the form of almond milk. The added advantage of almond milk is the presence of antioxidants. It provides monounsaturated fatty acids (67 %), dietary fiber (13.2 %), specifically an essential amino acid arginine (25 %), and essential minerals such as magnesium, copper, phosphorus, and potassium. It is rather low in protein amounts than other plant milks (Sunidhi et al., 2021). It contains bioactive compounds, mainly vitamin E (6.33 mg/100 g), flavonoids, and polyphenols (Nissen, di Carlo, & Gianotti, 2020). Peanut and almond milk provide notable amounts of vitamin C and E. An 8-oz serving of almond milk provides 40 cal, 1.51 g of protein, and 3.58 g of fat. Cashew milk can meet 20 % RDA of magnesium (Sunidhi et al., 2021).

**Fig. 2:**
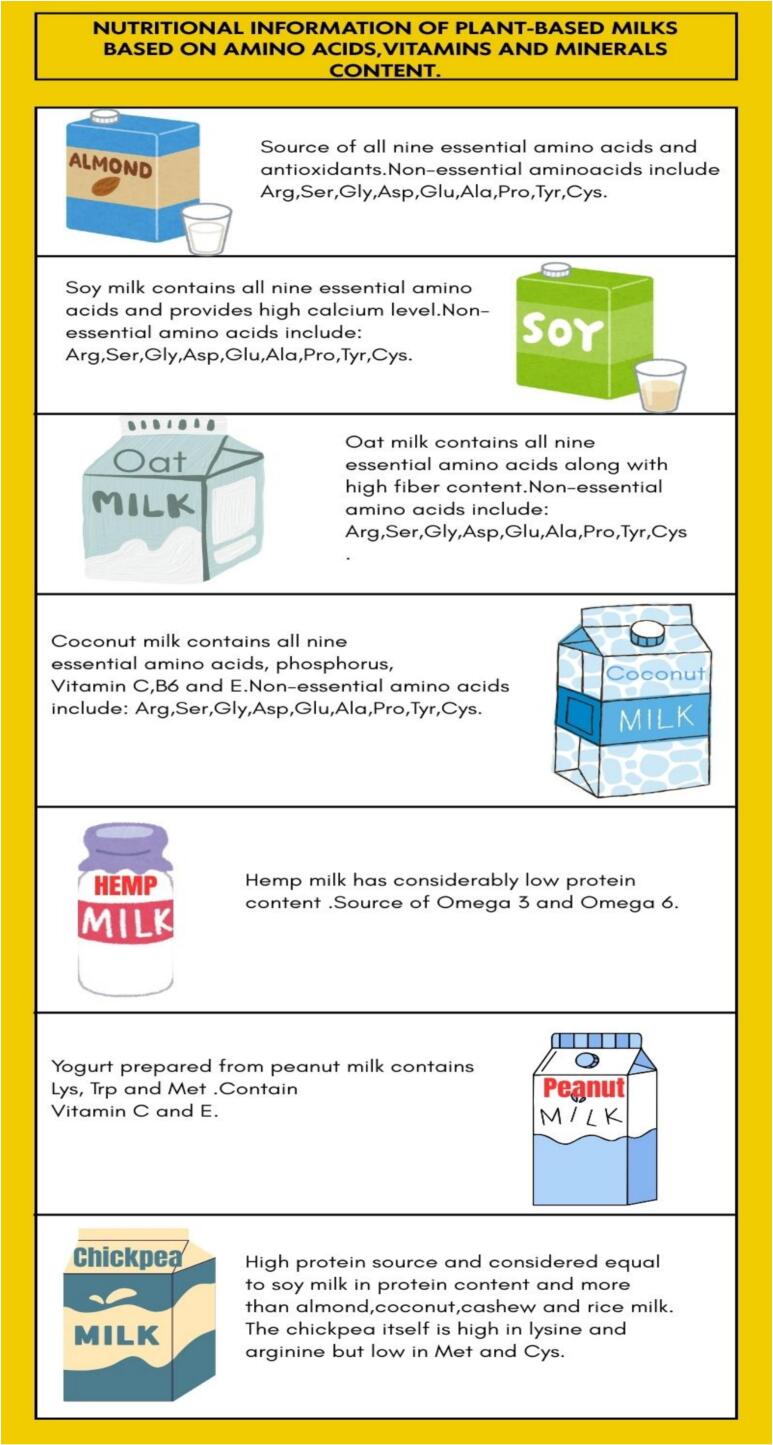
Nutritional Information of Plant Based Milk Alternatives based on Amino Acids, Vitamins and Minerals Content.

From the seeds category, hemp seed is a substitute for milk. It contains 20–25 % protein content, a good proportion of omega-6 and omega-3 fatty acids, and a lipid content of 21.08 g/100 g. Some essential minerals and vitamins are copper, magnesium, calcium, and phosphorus (Leahu, Ropciuc, & Ghinea, 2022). It offers high amounts of protein and meets the 50 % RDA of alpha-linolenic acid. It yields 10 essential amino acids (Sunidhi et al., 2021). An 8-oz glass of oat milk contains 130 cal, 2 g of fat, and 4 g of protein. It has a high amount of fiber and is an allergen-free option as it does not contain soy, nuts, or legumes. “It is a safe option for those with Celiac disease if made with certified gluten-free oats” (Sunidhi et al., 2021). Coconut milk provides a good profile of essential amino acids and has 3.5–4 % of protein, especially globulins and albumins, and 31–35 % of fat content, mainly comprising medium-chain fatty acids. It also contains some important minerals and vitamins such as calcium, phosphorus, vitamins C, B_6,_ and E (Pachekrepapol et al., 2021). Some of the major nutrients present in PBMAs are shown in the Fig. 2.

As far as health and nutrition are concerned, knowing only the amino acid profile of a food is not enough. For this reason, in 1991, FAO and WHO pointed out that the quality of protein is important as it is related to how well absorption of amino acids takes place in the human gut. For example, there are certain inhibitors present in soy milk that affect protein digestibility. These inhibitors can only be removed with high-heat treatment. This adds an extra processing step. There are two scores named as Protein Digestibility-Corrected Amino Acid Score (PDCAAS) and the Digestible Indispensable Amino Acid Score (DIAAS). The latter one is more recent and recommended to determine protein quality as it measures the individual digestibility of each amino acid. Plant-based beverages have low protein quality, as shown by these scores. The DIAAS scores for soy, coconut, oat, and almond-based drinks are 1.08, 0.72, 0.59, and 0.43, respectively. The cow milk score is 1.45. The oat, coconut, and soy drinks provide a better protein score than almond beverages when using the PDCAAS score. The soy-based beverages provide protein comparable to cow milk but still have a very low quantity of methionine. So, overall, plant-based milks need a lot of combinations or large quantities of amino acids to compete with cow milk protein content. For this reason, especially, vulnerable groups of the population like infants, children, the elderly, and vegan diet followers must be given special attention in regard to essential amino acids intake.(Moore et al., 2023).

Similarly, plant-based milk will have low protein levels even after combinations of different plant proteins. This is evident from PDCAAS and DIAAS scores that almond milk has low levels of methionine and cysteine, whereas soy protein isolates have shown higher levels of methionine, cysteine, and lysine. (Chalupa-Krebzdak, Long, & Bohrer, 2018). Hemp milk has considerably low protein content but acts as a source of Omega-3 and Omega-6 (Beşir et al., 2022). Yogurt prepared from peanut milk contains lysine, tryptophan, and methionine, and acts as a source of Vitamin C and E (Zhang et al., 2024). The chickpea milk is high high-protein source and considered equal to soy milk in protein content and more than almond, coconut, cashew, and rice milk. The chickpea itself is high in lysine and arginine but low in methionine and cysteine (Hamioud, 2025).

A research study was conducted by (Jaeger, Dupas de Matos, Frempomaa Oduro, & Hort, 2024) in New Zealand on 143 participants and 18 PBMA samples. An exploratory analysis was done to check the correlation between energy, protein, fat, and carbohydrate content, but it showed no significant difference among them. However, sugar showed a positive association. Milk with a higher sugar content was preferred, and the product with higher levels of sodium was least preferred by the consumers.

A study was conducted by (Walther et al., 2022) in Switzerland to compare the nutritional composition of 27 plant-based dairy alternatives and 2 cow milk samples. This study included plant milk extracted from almonds, cashews, coconut, hemp, oat, rice, soy, and spelt. The method used to check the solid content was dry heating using an oven at 102 °C. The protein content was analyzed by the Kjeldahl method, and the results showed that, from all 27 samples, soy milk showed the highest content of protein. PBMA had a lower content of glucose and a higher content of sucrose as compared to dairy milk and vice versa. Starch content was also found in the PBMAs, but milk has no traces of starch. The highest amount of fat content was found in cashew milk, followed by almond and soy milk. The vitamin content was higher in conventional milk and relatively low in PBMAs; the only source of high content in some products was due to the fortification (Walther et al., 2022).

The energy present in almond milk was 21.8 kcal/100 mL, 45.4 kcal/100 mL in oat milk, and soy milk had 37.8 kcal/100 mL, whereas dairy milk provided the highest energy of 53.1 kcal/100 mL. The mineral requirements were well met by PBMAs (PBMAs) because of supplementation. Fat-soluble vitamins, especially D and E, were higher in soy-based milk; in contrast, water-soluble vitamins were lower in PBMAs as compared to cow milk. Milk alternatives made from legumes have a protein content like that of cow's milk. Like cow milk, PBMAs also pose allergen threats, such as gluten from oats, tree nuts, and soy sources (Paul et al., 2020).

Jemaa et al. (2021) researched four types of milk alternatives: soy, almond, hemp, and quinoa. They were prepared in the laboratory due to their unavailability in the market. Upon checking the macronutrients and micronutrients of these milks, the oat milk had the highest content of carbohydrates, 23 g/100 mL, then 7 g/100 mL for hemp seed milk, 6 g/100 mL of carbohydrates in almond milk, and the least in quinoa milk, which is 3.7 g/100 mL. In a nutshell, the content of carbohydrates in hemp, almond, and oats was higher than that of cow's milk. For the protein content, oat milk led the way with 5 g/100 mL of protein, then hemp milk with 3 g/100 mL of protein, followed by almond milk that contains 2 g/100 mL, and the least in quinoa, which contains only 1.5 g/100 mL of protein. The protein content of cow's milk is 3 g/100 mL, so hemp and cow's milk have the same protein content, whereas oat milk has more protein than cow's milk. The lipid content is the highest in hemp seed milk, i.e., 7 g/100 mL, 2.7 g/100 mL in almond milk, and 1.8 g/100 mL in quinoa milk. The fat content of cow's milk is 3.63 g/100 mL; the hemp seed milk surpasses cow's milk in fat content. All four showed higher amounts of calcium than cow's milk (Jemaa et al., 2021).

A survey carried out by (Angelino et al., 2020), stated that 77 % of the plant-based beverages were labeled organic, but they had more carbohydrate and sugar content than non-organic ones. Through this survey, it can be concluded that organic certifications are not a reliable marker of better nutrition quality.

To meet the increasing demand for PBMAs, new blends and new plant beverages are being manufactured. Some of them meet some criteria of nutrition, whereas some lack them. Fortification and supplementation are done to overcome the low amount of nutrients. Each class provides different nutrients based on the amount, processing, and type used. It's a substitute for cow's milk, not a replacement; a balanced diet should be consumed with them to overcome deficiencies. Nutrition labels should be read carefully to understand the constituents of the milk.

## 5 Improvement in shelf life

Soymilk was supplemented with curcumin, a natural bioactive present in turmeric that has many health benefits, as it provides anti-viral effects, protects from viruses, and acts as a preventive agent against many diseases. A study was conducted to develop soy milk with added curcumin to enhance its physical, chemical, antioxidant, and technological profile. The substitution of 15 % of turmeric juice was preferred in terms of organoleptic properties. The addition of turmeric juice reduced the separation of the soy milk during storage, causing it to remain stable for 20 days under refrigeration conditions (Basha, Kaur, Kaur, & Singh, 2024). Research conducted by Zheng, Zhou, and McClements (2021) incorporated curcumin in many plant-based milks such as almond, cashew, coconut, and oat milk alternatives. The incorporation was done through the methods of pH-driven strategy, and it increased the bio accessibility of curcumin in all milk analogs.

Recent years have seen an increasing trend to fortify PBMAs with antioxidant and nutraceutical compounds to deliver added health benefits and to extend shelf life. Several bioactive classes are particularly promising for PBMA formulation: mangiferin (a xanthone glycoside) with strong antioxidant and antimicrobial potential; ginger-derived phenolics (gingerols, shogaols) that provide antioxidative, anti-inflammatory, and flavor-modulating effects; and natural colorants with concomitant antioxidant activity (for example, carminic acid and other pigments), which can replace synthetic dyes while contributing functional value. Incorporation of these biomolecules into beverage matrices demands attention to extraction and purification (to remove co-extracted impurities), choice of delivery system (emulsions, micro/nanoencapsulation, or complexation) to improve solubility and bioaccessibility, and compatibility with thermal or non-thermal processing to avoid degradation.

Mangiferin has been utilized in many functional foods to improve the nutritional profile as it provides various health benefits such as radioprotective, anti-diabetic, antioxidant (Castro-Muñoz, Cabezas, & Plata-Gryl, 2024). Though this study did not provide any addition of mangiferin in any plant-based milk but it can be studied to develop a plant-based milk alternative with added mangiferin (Castro-Muñoz et al., 2024). Like Mangiferin, another very beneficial bioactive compound known as gingerol extracted from ginger, possesses many health benefits, and can also be utilized to develop a new functional product or a nutraceutical (Garza-Cadena et al., 2023).

Recent comprehensive reviews summarize optimized extraction and purification workflows and highlight progress towards food-grade, scalable processes for these compounds — including mangiferin extraction and food applications, carminic acid production and purification as a natural food colorant, advanced strategies for isolating ginger bioactives and polishing gingerol fractions, and up-to-date approaches for ginkgo-derived terpenoids that illustrate generalizable purification strategies for sensitive nutraceuticals. These studies support the feasibility of producing fortified PBMAs that combine improved oxidative stability, sensory appeal, and added nutraceutical value, while also emphasizing the need for formulation work to ensure stability, regulatory compliance, and validated bioavailability. Some new technologies are being introduced in the industry to solve the challenges related to physicochemical characteristics, nutritional qualities, stability, and extended shelf life. The new technologies include pulsed electric field (PEF), cold atmospheric plasma (CAP), ultrasound (US), ultra-high-pressure homogenization (UHPH), ultraviolet C (UVC) irradiation, ozone (O3), and hurdle technology used in PMA formulations (Mehany et al., 2024).

## 6 Health effects of PBMA

For a long time, milk from cows has been an essential nutrient component of human health due to the presence of many nutrients like carbohydrates, proteins, fats, minerals, etc. (Reyes-Jurado et al., 2023). Since 2012, a decline in the consumption of cow's milk has been noticed because of its inability to be digested and absorbed properly (Silva et al., 2020). The major problems people face from Cow's milk or other dairy products are their saturated fats, high sugar levels, lactose intolerance, hormonal content from injecting cattle for fast growth, protein allergies, and misuse of antibiotics in the cattle industry (Kaskous, 2021). These practices caused an increase in the consumption of plant-based diets, which include vegetables, pulses, fruits, legumes, seeds, and cereals, due to many motives, including mindsets of environmentally friendly approaches, loathing the cruelty towards animals, and ensuring a healthy lifestyle. Hence, the demand for plant-based dairy alternatives has increased to 61 % since 2012 (Reyes-Jurado et al., 2023). All these nuts, cereals, and their oils have limitless benefits for the health of individuals as they are rich in phytochemicals, bioactive compounds, and other macro and micronutrients (Aydar et al., 2020) and are free from lactose or cholesterol, as both of these are only present in products of animal origin (Ebabhi & Adebayo, 2022). By knowing the nutritional value of PBMAs, people have faith that they can improve their health and help them achieve their well-being goals (Reyes-Jurado et al., 2023).

PBMAs have many positive effects on human health, but they lack a lot of essential nutrients (Table 2). Besides the allergens, PBMAs are lower in protein than dairy milk, causing a deficiency if one replaces cow's milk with PBMAs for a protein source. Also, many anti-nutrients are present in plant-based milk, which inhibit the absorption of nutrients, reduce digestion and utilization, and have adverse effects on the human body. Proper processing is required to reduce or eliminate these anti-nutritional factors (Aydar et al., 2020a, 2020b).

**Table 2:** Pros and Cons of Consumption of Plant-Based Milk Alternatives (Reyes-Jurado et al., 2023; Sethi et al., 2016).

Advantages	Disadvantages
No presence of cholesterol No protein of cow's milk Rich in fiber Lactose free Rich in low saturated fats Presence of isoflavones	Allergies by other proteins Less protein contents Short in micronutrients Less acceptable by consumers Have antinutrients (trypsin inhibitors, inositol phosphate, phytic acid)

There is a growing global demand for a transition towards a sustainable, equitable, and resilient food system, which has accelerated interest in PBMAs as substitutes for dairy milk. From a nutritional standpoint, PBMA's are characterized by the absence of cholesterol and a lipid profile predominantly composed of monounsaturated and polyunsaturated fatty acids, with relatively low levels of saturated fatty acids, except in coconut-based formulations. Additionally, the lipid fraction of PBMA's contains bioactive components, including essential fatty acids (EFAs) and medium-chain fatty acids (MCFAs), which are associated with recognized health-promoting effects and contribute to their appeal among health-conscious consumers.

Beyond nutritional attributes, the increasing prevalence of lactose intolerance and milk protein allergies, along with environmental concerns related to conventional dairy production and ethical considerations associated with animal welfare, has further driven consumer interest in PBMA's, particularly in regions such as North America, Europe, and East Asia. Consequently, the global PBMA market has experienced rapid expansion and is projected to continue growing substantially in the coming years. Despite this progress, recent reviews highlight the need for continued research to address existing challenges related to protein quality, micronutrient fortification, allergenicity, sensory properties, and long-term health outcomes, in order to ensure that PBMAs can effectively meet consumer expectations for sustainable and nutritionally balanced food products (Antunes et al., 2024; Blasi et al., 2023; Mantzourani et al., 2025).

### 6.1 Health effects of almond milk

Almonds are a nutrient-dense food, and due to their rich nutritional profile, they are being used as a base to make milk alternatives. That's why they are gaining popularity in European markets as a cow milk alternative (Mukherjee, Tewari, & Pramanik, 2022). Almonds are rich in soluble sugars, proteins, lipids, minerals, fibers, and other nutrients like zinc, potassium, selenium, copper, and phosphorus. Almonds also show a great potential to be a prebiotic because of the presence of arabinose (Ozcan, 2023). Most of the bioactive compounds that are present in almond milk are beta-sitosterol, folate, campesterol, vitamin E, mainly tocopherol, vitamin B complex, sigmasterol folate, flavonoids, phytosterols, etc. (Mukherjee et al., 2022). The milk from almonds proves to be a very powerful source of antioxidants. It is also a low-calorie drink that helps in lowering low-density lipids in plasma, is good for the health of the gastrointestinal tract, and helps prevent anemia (Sobhy, El Abd, Elsabie, & FathyForsan, 2021).

In addition to health benefits, they also pose some health issues. Some allergies have been reported due to almond milk because of some potential protein compounds such as 2S albumin, conglutin γ, and amandin. Cystine and methionine are naturally present in almonds as limiting amino acids, making them a poor source of these essential amino acids. Almond also exhibits anti-carcinogenic activity, which is affected in the presence of sucrose (Gonçalves et al., 2023).

### 6.2 Health effects of sesame seed milk

Milk from sesame seeds proves to be a good source of minerals and proteins and is known for its appreciable activity as an antioxidant (Vijaya & Nirmala, 2021). The bioactive compounds present in sesame milk are lignans such as sesamin, sesaminol, sesamolin, etc. Sesame milk is an excellent antioxidant and prevents cell damage caused by oxidation. It also protects against various carcinogens, reduces tumors, and viral activities (Reyes-Jurado et al., 2023).

Sesame milk contains lysine, a limiting amino acid, and anti-nutritional components like phytates and oxalates, which reduce the bioavailability of calcium and many other essential nutrients in the body (Jasim, 2021). Sesame seeds are a rich source of protein, but the processing conditions denature protein, resulting in a low protein content in sesame milk (Silva et al., 2020).

### 6.3 Health effects of coconut milk

Coconut milk is extensively used as a milk beverage and as an ingredient in many recipes in Southeast Asia (Gengan, 2025). It has a high fat content and is used as a thickener in many curries. Lauric acid and vitamin E are the bioactive components of coconut milk. They provide numerous health benefits, including efficient brain development, boosting immunity, and improving the elasticity of blood vessels. Coconut milk also contains triglycerides, which help in weight loss, prevent aging, and promote skin nourishment (Gengan, 2025). Coconut milk contains saturated fats like lauric acid, caprylic acid, and capric acid (Hewlings, 2020). It is deficient in monounsaturated and polyunsaturated fatty acids, which can be harmful as they increase low-density lipoproteins and bad cholesterol. Coconut milk is a poor source of calcium, and a diet rich in coconut milk can increase the risk of fractured or broken bones.

### 6.4 Health effects of soy milk

Soy milk is known for its freshness and good nutritional composition. It is an inexpensive beverage with high concentrations of monounsaturated and polyunsaturated fatty acids, making it good for the health of the cardiovascular system (Paul et al., 2020). It has isoflavones which are effective against osteoporosis, heart diseases, and cancers (Paul et al., 2020). Soy milk contains excellent amounts of bioactive compounds like genistein, glycitein, and daidzein, along with fiber, iron, calcium, zinc, vitamin B, etc. The protein content (2.36–8.71 %) of soy milk is comparable to that of dairy milk and is the highest among all the PBMAs. Due to its bioactive constituents, soy milk is effective in reducing blood pressure, maintaining blood lipid levels, and preventing chronic diseases. It is also beneficial for osteoporosis as it enhances bone density and reduces the rate of fractures. Soy-based beverages fermented by *Lactobacillus acidophilus* also help in lowering the levels of low-density lipoproteins, cholesterol, triglycerides, and glucose in the liver and serum, along with an improvement in antioxidant levels in blood serum. (Tiss et al., 2020).

Soybeans contain oligosaccharides like raffinose, stachyose, and sucrose. α-Galactosidase is an enzyme that breaks down the galactosidase bonds, helping in the digestion of oligosaccharides. Conversely, α-galactosidase is not present in the human intestinal tract, and the oligosaccharides remain indigestible, causing flatulence and gas. Eating soy-rich foods upsets the hormonal balance, reduces testosterone production in men, and affects the gestation period and fertility in women. These foods adversely affect the brain, ovaries, and mammary glands and cause cancer of the reproductive tract (Paul et al., 2020).

### 6.5 Health effects of peanut milk

Peanut milk has huge nutritional importance as peanuts are rich in minerals, proteins, and fatty acids like oleic acid and linoleic acid, which are valuable for the nutrition of humans. Niacin, arginine, vitamin E, and resveratrol are some bioactive components of peanut milk (Paul et al., 2020). These bioactive compounds prevent oxidative damage and reduce the risk of coronary heart disease, stroke, and cancer (Gengan et al., 2025) and improve the function of the digestive system.

### 6.6 Health effects of oat milk

Oat milk has a mildly sweet taste and is widely used in cereals, smoothies, soups, and curries. Among all plant-based milks, oat milk has the highest fiber content, which makes it effective for improving digestion and reducing cholesterol levels. The bioactive compounds present in oat milk are avenacosides A and B, β-glucan, α-tocopherols (Hu et al., 2023), α-tocotrienol, and avenanthramides, etc. Oat milk helps maintain blood glucose levels, reduces cholesterol levels, has high satiety levels, and shows antipathogenic effects, keeping humans safe from many diseases. On the other hand, oat milk, as compared to dairy milk, is sparse in proteins, minerals, and calcium, and can contain a potential allergen that can cause hazards to human health (Sunidhi et al., 2021). Oat milk contains lysine, a limiting amino acid, and several anti-nutrient components like inhibitors of trypsin and phytates, that decrease the efficiency of nutrient absorption, leading to nutritional deficiencies (Basinskiene & Cizeikiene, 2020).

Recent changes in lifestyle are associated with a change in diet and consumption of food. In relation to this change, demand for PBMAs as a substitute for dairy milk has increased. PBMAs have lower unsaturated fatty acid content as compared to conventional milk and do not pose health risks such as allergies or intolerances that conventional milk does (Leahu et al., 2022). Plant milk can provide many health benefits, but it is inferior to cow's milk in many ways, as it provides lower protein, vitamins, minerals, and energy in comparison to dairy milk (Craig et al., 2023). Also, the processing and formation of milk from plants reduces their nutritional content, making them a poor source of nutrients. Many consumers have inadequate information regarding the appropriate intake of plant-based milk products. So the consumers should not completely shift towards plant-based substitutes as these products are not comparable to dairy milk in terms of chemical and nutritional composition (Reyes-Jurado et al., 2023).

## 7 Allergens

Allergens are specific components (mostly proteins or haptens) that trigger the response of immune cells, leading to immunological reactions with adverse health effects (Reyes-Jurado et al., 2023). Protein has the potential to cause an allergy by triggering the sensitive immune system and causing IgE-type reactions (El Mecherfi et al., 2020). Some consumers replace dairy milk with plant milk to avoid cases of intolerance and allergies. However, PBMAs are also associated with cases of allergic reactions, but there is a lack of data as these instances are not documented (El Mecherfi et al., 2020). Many plant milks have complicated nutritional labels, as they contain several number of ingredients, making it difficult for consumers to understand and interpret them.

**Fig. 3:**
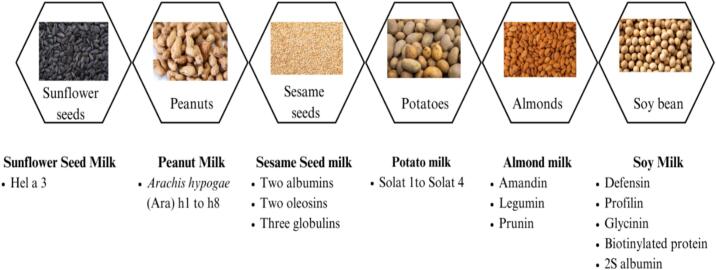
Different types of Plant Based Milk Alternatives and their potent allergens.

Milk from nuts and soy is abundant in potential allergens, and they are most often associated with anaphylaxis (Reyes-Jurado et al., 2023). Soybean proteins have toxic reactions that can be IgE-dependent or independent, most commonly occurring in children with severe symptoms of the intestine and skin, which can even lead to severe enterocolitis (Munasir & Sekartini, 2020). Some of the allergens present in soybeans are defensin, profilin, glycinin, seed biotinylated protein, and 2S albumin. Allergic reactions from almonds are quite common, with symptoms ranging from gastrointestinal disturbance to irritation of the respiratory and cutaneous areas (Bezerra, Ribeiro, & Igrejas, 2021) and anaphylaxis (Reyes-Jurado et al., 2023). The important allergenic compounds present in almond milk are amandin (protein), legumin, and prunin (major seed protein) (Paul et al., 2020). In the peanut milk industry, industrialists face many issues regarding peanut allergy. There are no preventative measures to avoid peanut allergy; however, a LEAP study shows that early exposure of high-risk infants to peanut proteins may be protective against allergy development. The symptoms of peanut allergy may vary from individual to individual and can include rashes, morbilliform, flushing, vomiting, nausea, pain in the abdomen, sore throat, wheezing, cough, sneezing, along with cardiovascular collapse and anaphylaxis. Sesame milk is also associated with allergic reactions as it contains eight allergens, two albumins, two oleosins, and three globulins (Oriel, Elizur, & Sicherer, 2024). Symptoms of sesame allergy include vomiting, coughing, urticaria, etc. (Oriel et al., 2024). Conlinin allergen is found in flax seeds, which can cause severe anaphylaxis leading to death (Mota, Vasconcelos, Bartolomé-Zavala, Silva, & Coimbra, 2024). Numerous allergens have been detected in coconut milk, but only one allergen has been registered with the official allergen nomenclature subcommittee of the International Union of Immunological Societies. This allergen is a vicilin-like protein (53 kDa), and its symptoms include loss of consciousness, urticaria, difficulty breathing, vomiting, anaphylaxis, angioedema, etc. (Iddagoda et al., 2022). Major allergens present in PBMAs are represented in Fig. 3. Fig. 3 provides a comparative overview of commonly used plant sources for PBMAs and their dominant allergenic proteins. Oilseeds and legumes such as peanuts, soybeans, sesame seeds, and almonds contain storage and defense proteins (e.g., Ara h proteins, 2S albumins, legumin, vicilin, profilin) that are often resistant to thermal processing. Potato- and sunflower-based milks contain allergens such as patatin and Hel a 3, respectively, which may persist depending on extraction and stabilization conditions.

**Fig. 4:**
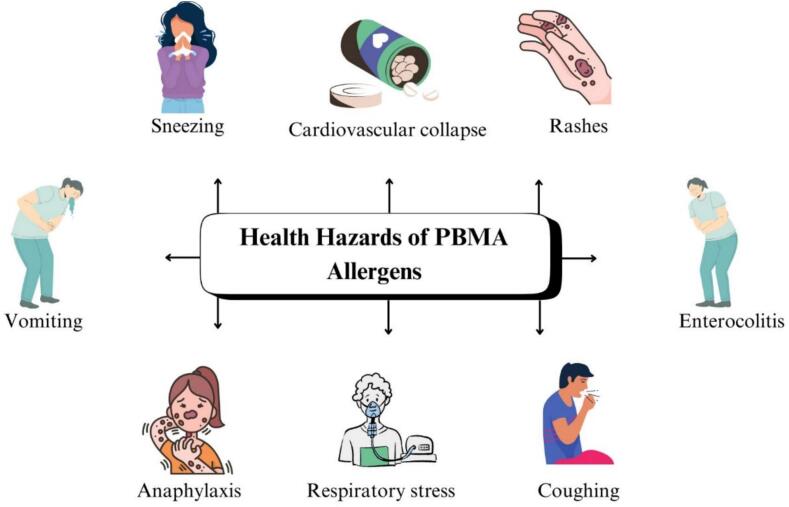
Common health hazards associated with Plant Based Milk Alternative allergens.

Common health hazards associated with PBMA, and their potent allergens, are shown in Fig. 4. Fig. 4 illustrates not only the clinical manifestations associated with PBMA allergens but also their physiological basis. Ingestion of allergenic proteins may trigger IgE-mediated immune responses, leading to mast cell degranulation and histamine release. This cascade results in respiratory symptoms (sneezing, coughing, respiratory distress), gastrointestinal disturbances (vomiting, enterocolitis), dermatological reactions (rashes, urticaria), and systemic effects such as cardiovascular collapse and anaphylaxis. The figure emphasizes that the severity of symptoms depends on allergen type, protein stability, individual sensitivity, and the degree of processing-induced protein modification.

### 7.1 Mitigation and structural modification of allergenic proteins in plant-based milk alternative

The dense and complex tertiary and quaternary structures of plant proteins are primarily responsible for IgE-mediated allergic responses. However, specific processing treatments can denature these proteins, resulting in partial reduction or removal of allergenic epitopes. Thermal treatments such as blanching, boiling, steaming, pasteurization, and autoclaving are widely reported to reduce IgE-binding capacity by disrupting protein conformation (Costa et al., 2022; Costa et al., 2022). Nevertheless, several plant allergens remain stable under extreme conditions; for instance, Costa, Bavaro, et al. (2022) and Costa, Villa, et al. (2022) demonstrated that high-pressure processing does not completely eliminate allergenic risk, as structural alterations may be insufficient to fully suppress immunoreactivity.

Non-thermal technologies have gained attention as complementary approaches. Cold plasma treatment has been shown to reduce peanut allergens Ara h 1 and Ara h 2 by approximately 65 % (Venkataratnam, Cahill, Sarangapani, Cullen, & Barry-Ryan, 2020). Structural modifications leading to reduced allergenicity are typically associated with disruption of secondary and tertiary protein structures. However, legumins present in peanuts and soybeans exhibit notable resistance, maintaining allergenic potential even after processing. In contrast, allergens such as gliadin and profilin display reduced IgE reactivity following loss of their three-dimensional structure, unlike 2S albumins and non-specific lipid transfer proteins, which are relatively heat stable (Daryani et al., 2024).

Additional allergen-mitigation strategies include alkylation, reduction, and enzymatic hydrolysis. Heat treatment has been shown to significantly reduce the allergen Cor a 1 in hazelnut, while enzymatic proteolysis effectively cleaves allergenic proteins into smaller, less immunoreactive peptides (Daryani et al., 2024). Costa, Bavaro, et al. (2022) and Costa, Villa, et al. (2022) further emphasized that thermal processing methods such as blanching, autoclaving, steaming, pasteurization, and boiling reduce IgE-binding capacity by disrupting allergen secondary structures. In peanut extracts treated at 138 °C for 30 min, substantial reductions in allergenic activity were observed, with enzymatic treatment and cold plasma reducing Ara h 1 and Ara h 2 by up to 95 % and 65 %, respectively (Daryani et al., 2024).

In almond milk processing, operations such as roasting, blanching, crushing, and oil extraction influence allergen stability. The major almond allergen Pru du 6 is highly resistant to thermal treatments but shows significant sensitivity to enzymatic digestion, particularly by pepsin. Fermentation-based mechanical and biochemical treatments further contribute to allergen reduction (Paul et al., 2020). Similarly, allergenic proteins in legume- and bean-based milks are reduced through solid- and liquid-state fermentation using *Bacillus subtilis*, which also enhances antioxidant activity. Filtration during peanut milk processing contributes to partial removal of allergen-rich fractions (Daryani et al., 2024).

Despite the effectiveness of these strategies, it is important to recognize that most treatments only reduce allergenicity rather than completely eliminating allergenic risk. Therefore, individuals with known food allergies should exercise caution, and appropriate allergen labeling remains essential for consumer safety.

## 8 International market trends

Innovative food and drink markets like PBMAs are growing rapidly worldwide (Sunidhi et al., 2021). There has been an increased liking for PBMAs (PBMAs) among consumers, resulting in a rise in their market growth. PBMAs constitute a rapidly growing market within the food industry. Its market value is expected to increase by 11.5 % by 2023 in comparison to $17 billion in 2018. Soy milk in China also shares the cultural history of PBMAs (Moss et al., 2022). Worldwide sales of PBMAs have doubled between 2009 and 2015. In the U.S., the sales of PBMAs increased 9 % to USD 1.6 billion in the first half of 2018. According to a report, the market of non-dairy milk alternatives reported total retail sales of USD 6 billion in the U.S. It is estimated that the industry will reach USD 28 billion in total retail sales in the U.S. (Silva et al., 2020). The PBMAs market is also expected to grow in the Middle East, Africa, and South America (Adamczyk, Jaworska, Affeltowicz, & Maison, 2022). An overview of major global companies producing PBMAs, along with their formulation strategies, consumer acceptability, and economic considerations, is presented in Table 3.

**Table 3:** Global plant-based milk alternative producers, key formulations, consumer acceptability, and economic aspects of the current market.

Company (brand)	Flagship PBMA products/categories	Key formulation/features (fortification, bioactives, texture)	Consumer acceptability/positioning	Economic/market notes
Oatly	Oat drinks (barista, original, light, protein variants)	Oat-based, barista foaming blends, some protein-enriched launches; premium positioning for coffee sector	High acceptability for taste and coffee use; strong brand recognition in Europe and North America.	Leading oat-milk specialist; rapid revenue growth in recent years but faces regional volatility; strong share in oat segment.
Danone (Alpro, Silk, Other PB brands)	Alpro (oat, soy, almond), Silk (US: soy/almond/oat)	Wide portfolio: fortification (Ca, vit D), barista blends, protein/low-sugar variants; large R&D and formulation capability	Broad mainstream positioning across retail and foodservice; strong distribution networks.	Danone owns Alpro/Silk (completed acquisition via WhiteWave); major multinational player investing in plant-based expansion.
Blue Diamond (Almond Breeze)	Almond Breeze almond milk (Original, Unsweetened, Barista)	Almond-based, fortification with calcium/vitamins; barista/creamer lines; value and premium SKUs	One of the best-known almond brands; strong acceptability among almond-milk buyers.	Blue Diamond (cooperative) is a leading almond milk brand; strong presence in almond segment and category promotions.
Califia Farms	Almond, oat, blended milks; ‘Complete’ high-protein launch	Product innovation (pea/chickpea protein blends, protein-complete variants), premium positioning and clean-label claims	Well rated for creaminess and formulation innovation (barista, creamers); premium niche with good consumer acceptance.	Independent/scale-up brand focused on North American retail and foodservice; invests in fortified, higher-protein formulations.
Vitasoy	Soy, almond and other plant milks (Asia-focused)	Fortified products (vitamins/minerals), regionally tailored flavors; high emphasis on nutrition criteria	Strong acceptance in Asia (China, Hong Kong); mainstream market leader in several Asian markets.	Large Asia-Pacific player with steady revenue; sustainability and nutrition reports highlight product development.
Elmhurst/Others (small innovators)	Elmhurst 1925 (nut/oat/pea blends), local/startups	Clean-label, high-protein or minimal-ingredient lines; niche functional offerings (fermented, enzymatic treatments)	Valued by consumers seeking fewer ingredients or craft formulations; limited national penetration vs. majors.	Many smaller innovators push novel formulations (pea blends, fermented milks, microencapsulated bioactives); often target premium channels and foodservice.

A variety of milk alternatives of plant origin are available in the commercial global market. The PBMAs market in the United States is showing fast-paced growth, which is evident from the yearly growth of around 1.8 billion USD. Globally, the compound annual growth rate for PBMA is greater than 10 % and by 2023, it is predicted to be more than 26 billion USD. The increased preference for PBMAs is due to multiple reasons, including medical conditions like lactose intolerance and allergies, hormones, and cholesterol associated with animal-sourced milk. Marketing strategies that promote plant-based products as healthy alternatives, along with animal well-being, environmental sustainability, and vegan food trends, have influenced consumers' choices. As a result, different food-based industries are also introducing PBMAs to increase variety in their products (Pointke, Albrecht, et al., 2022a, 2022b).

The demand for PBMAs is growing. At present, the world international market for these products is becoming a huge business, which is expected to reach 26 billion dollars in the next five years. Most consumers who prefer PBMA are concerned about their environment, health, and diet. A variety of products are now available based on nuts, seeds, or beans. With progress in advancements, a huge variety of balanced and naturally made PBMAs is expected soon. Still, there are problems related to health and taste, which reduce the interest of consumers. Mixed-culture fermentation is a method that can improve the sensory and nutritional properties of these products. Previous studies have concluded that mixed-culture fermentation depends on the type of microorganism being used. Due to the lack of knowledge regarding their modes of interaction, microorganisms are selected on an experimental basis. Effective selection and combination of micro-organisms can ensure efficient fermentation, producing products of good taste and quality (Pointke et al., 2022).

In the USA, a decline of 22 % in animal milk consumption has been recorded for the period 2000–2016. In the U.S., the market share of PBMAs increased even when animal milk consumption was prevalent. The PBMAs market is growing at a rate of 8 % and by 2024, it is expected to reach USD 25 billion. A decline in consumer demand for dairy milk has reduced the overall dairy farm income. A loss of 3.21 USD per hundredweight of milk was reported for the year 2018 in the U.S. From 2018 to 2019, almost 2500 dairy farms went out of business, and dairy cow stocks also decreased by almost 100,000 (Boaitey & and Minegishi, 2020). The results of a report have shown that there is a need for production and marketing of PBMA in Europe due to consumer interest and demand (Vaikma et al., 2021).

In Slovakia, high prices of dairy milk and the availability of PBMAs that taste good have decreased the usage of dairy milk by 1.88 kg per capita. The plant-based milk analogs hold a huge market segment, and their sales are worth 1.8 billion USD. The U.S. and Canada are the two countries with the highest sales of PBMAs. The sales of plant milk grew by 61 % from 2012 to 2017, resulting in a 15 % decline in the sales of dairy milk. As per market predictions, the sales of PBMAs are expected to increase by 7.1 % per year and will reach 9.5 billion USD at the end of 2022. In 2014, the sales of PBMAs increased by 31 % in the USA, by 24 % in Europe, by 17 % in Latin America, and by 14 % in Asia-Pacific. In 2018, market growth of 16 billion USD was recorded for PBMAs globally; however, there was a decrease in sales of soy milk as compared to previous years. Among all plant-based milks, the lowest sales were made by rice milk, as it is mostly popular in Asia. Almond milk had the highest demand, and it is expected that the sales of almond milk will increase. The average sale of almond milk for 2013–2018 was calculated to be 287.14 million USD and is expected to increase to 3254 million USD in 2021. The market of PBMAs is expected to grow exponentially, as evidenced by the popularity of vegan lifestyles and plant farming in exchange for conventional animal breeding (Prytulska et al., 2021).

**Fig. 5:**
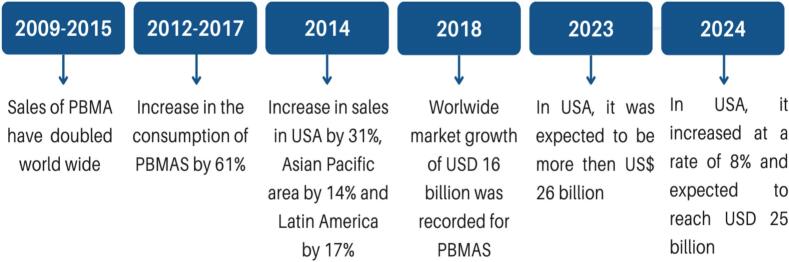
Graphical representation of market trends of Plant Based Milk Alternatives.

In the Western markets, the most popular PBMAs are soy-based drinks. A diverse range of plant-source drinks includes almond, rice, chickpea, sunflower seed, lupine, sesame, pea, coconut, and quinoa. PBMAs are becoming a household product, and in Germany, almost 50 brands are providing PBMA products that are available in supermarkets. According to a survey, 93 % of the consumers in Germany are buying PBMAs, which is higher than any other plant-based product. Oat milk is gaining popularity by leaving behind soy milk, and its demand is expected to increase in the future (Pointke, Ohlau, Risius, & Pawelzik, 2022). A graphical representation of market trends of PBMAs is shown in Fig. 5.

## 9 Consumer trends

Social concerns in terms of environmental sustainability, animal well-being, and health concerns have resulted in an increased number of consumers to choose PBMAs (PBMAs). Consumers who are compassionate about animals and the environment tend to select PBMAs instead of animal-sourced milk. Consumers with health conditions that limit their use of animal-based milk, such as high cholesterol levels, inability to digest lactose, or allergic response to milk protein, also adopt plant-based milk as an alternative (Moss et al., 2022). Most of the marketing strategies of PBMAs use terms like sustainability and environmental consciousness to convey that their products are free from animal cruelty and artificial additives. Marketing techniques for PBMAs suggest that plant-based milk products are similar to dairy milk, yet they can address the concerns of consumers regarding animal-sourced milk. As a result of the growing concerns among consumers about the environmental impact of dairy products, the dairy industry has started to opt for eco-friendly terms; for example, in 2018, 21 % of dairy products were marketed by using the term ‘grass-fed’. A survey report has shown that about 29 % of youth have sustainability concerns regarding milk produced from animal sources. Almost half of this consumer class is interested in buying products that are made from milk sourced from animals maintained in a nature-friendly environment (Schiano, Harwood, Gerard, & Drake, 2020).

Word association is a common method used to understand the thought process of consumers. It provides information that impacts the choice of consumers, like taste, health, and convenience, when purchasing food. Word association proves to be an effective method in comparison to closed questionnaires, as all the responses are spontaneously elicited, encouraging the consumers to share their thoughts freely (Moss et al., 2022). According to research, French and German consumers are more knowledgeable about PBMAs as compared to Polish consumers because PBMAs have a higher demand in Germany and France, whereas in Poland, consumers still prefer dairy products. Polish consumers failed to differentiate between dairy and non-dairy sources and considered cows as the only source of dairy products, whereas goat milk and sheep cheese were described as non-dairy products. Consumers in France mentioned more health benefits of PBMAs, like light in feel after use and low-fat content, especially in comparison to hard cheese. Another reason for consuming plant-based products was curiosity and trying various types of food, without completely avoiding dairy-based milk products. In Poland, marketing strategies have played a crucial role in developing the image of animal-based milk as the only healthy source. Studies in the U.S. have found that dietary habits play a vital role in ensuring that consumers utilize animal-based milk sources only. Those consumers who consumed dairy in their childhood were more willing to keep dairy in their diet as adults, in addition to giving dairy products to their children as well (Adamczyk et al., 2022).

Consumers' decision to like and buy food products is greatly influenced by how it feels and tastes. Even though PBMAs are gaining more and more interest, they still do not satisfy the sensory desires of many consumers. Some PBMAs, due to the presence of certain constituents (phenols, flavonoids), taste bitter, while others taste like beans and have a flavor similar to paint. PBMAs can change the taste of beverages like coffee, and many consumers prefer to add dairy milk instead of plant milk. The unacceptable taste of PBMAs can be the result of their long storage time, which affects fat and generates certain chemicals. To overcome the dry, powdery, and sand-like textures, flavors such as chocolate and vanilla are added to these products (Moss et al., 2022). Many studies have supported that PBMAs provide health benefits, another reason for consumers to choose plant-based milk. The level of knowledge regarding PBMAs is also different for consumers from different diet groups. Vegan consumers were more knowledgeable and considered that PBMA had fewer additives and a lower impact on the environment than their dairy counterparts (Pointke, Ohlau, et al., 2022).

Researchers often use the nine-point hedonic scale to find out if people like or dislike a certain food. Together with the hedonic scale, another method called check-all-that-apply (CATA) is also used. With CATA, people describe the food they are trying by looking at and choosing from the given list of qualities. This method is reliable when used with real consumers and has been used to study many different foods. Researchers also use the EsSense Profile to understand the feelings people have about food. The EsSense25 is a common questionnaire, used for lots of different kinds of foods. It includes asking people about their emotions when they eat different foods (Moss et al., 2022).

Most consumers perceive that PBMA has good taste and texture, and is beneficial for health and the environment. Certain consumers are also concerned about the ingredients of PBMAs and their overall cost. Almond and oat milk were the most liked PBMAs, as they had good sensory properties and mouthfeel. Organoleptic properties, such as bad aftertaste, non-white color, beans-like taste, and watery and strange flavors, were generally disliked by consumers (Moss et al., 2022). The unavailability of sufficient land resources, an increased trend for vegan and healthy lifestyles, insufficient dairy milk supply, fewer energy requirements, the presence of nutritional elements, and low levels of fats are some of the factors that have contributed to increased demand for PBMAs. A study reported that 90 % of people consuming PBMAs also consume cow milk, and taste is the main reason for choosing these alternatives (Silva et al., 2020).

## 10 Sustainability and environmental impact

Animal-based milk resources have been associated with several problems, namely, greenhouse gas emissions, land footprint, animal well-being, viral and pathogenic diseases, and increased antibiotic resistance (Moss et al., 2022). PBMAs have less environmental impact, including global warming potential, and water and land usage, than traditional animal-sourced products (Bryant, 2022). Soy milk has a lower carbon footprint in comparison to traditional cow milk, and hence, research suggests it to be an environmentally sustainable alternative to cow milk (Coluccia et al., 2022). In another study, life cycle assessment (LCA) methods were used to evaluate the environmental influence of PBMAs, as compared to dairy milk. In this study, PBMAs showed less impact on the envi onment. Among various types of dairy milk groups, UHT milk has limited environmental impact than that of fresh milk. Milk production through conventional methods is lower than the proportion of milk produced from grasslands. Almost 45 % of greenhouse gas (GHG) emissions take place from PBMAs even before they arrive at the processing facility. The production of greenhouse gases depends on the raw material used. Almonds have a higher global warming potential (GWP) as compared to oats, as they require greater quantities of nitrogen fertilizers, water, and electricity for irrigation. In Brazil, arable lands are cleared for soy production, whereas in Switzerland, only natural fertilizers are required. The packaging of PBMAs contributes towards 25 % of the global warming potential, while transportation of these drinks is responsible for 9 % of the total GWP. Regional transportation of plant-based milk through lorries produced an additional 5 % GWP for soy and 8 % for almonds. For international transport, almonds produced a further 4 % GWP. This data highlights that locally produced and processed plant-based products have lower levels of GHG emissions (Pointke, Albrecht, et al., 2022a, 2022b).

Several studies have determined that plant-based diets and dairy alternatives can minimize climate change. Conventional dairy beverages were responsible for the production of greenhouse gases, eutrophicating, acidifying, and ozone-depleting substances to a greater extent as compared to PBMAs (Carlsson Kanyama, Hedin, & Katzeff, 2021). A study related to dietary patterns and their environmental effects concluded that diets that include dairy milk have greater land and water requirements as compared to plant-based diets. Plant-based diets have overall fewer environmental indicators, including greenhouse emissions, in comparison to other food groups. Therefore, a change in diets from dairy and animal-based to plant-based food can considerably reduce overall greenhouse emissions (Carlsson Kanyama et al., 2021). Increasing evidence advocates a shift towards plant-based diets. A review article that analyzed land usage, life cycle, and evaluation models concluded that animal-based diets have a higher environmental impact than plant-based diets, as animal agriculture requires deforestation, freshwater, and contributes to GHG emissions and eutrophication (Bryant, 2022).

Environmental influence and resource utilization by food production can be reduced by efficient control. A study was conducted from 1964 to 2014 to determine the environmental impacts and resource usage by the California dairy production system for a period of 50 years. Carbon dioxide production significantly decreased by 45–46.9 % in 2014 as compared to 1964. A 55.7 % decrease in the enteric methane intensity was observed in 2014 as compared to 1964. Efficient crop strains and water management systems have resulted in the efficient use of water, resulting in reductions of water usage of 55–59 % in housing and milking, 88–90 % in crop production, and 52–54 % in the consumption of free water. An increase in crop yields has also reduced land usage by 89 % in 2014 as compared to 1964. Hence, according to this research, genetic, animal health, and nutrition improvements have resulted in higher milk production, which eventually reduced water and land requirements along with GHG emissions (Naranjo, Johnson, Rossow, & Kebreab, 2020). According to a report, North America is expected to utilize more plant-based products due to its dwindling natural resources and expanding PBMAs market (Craig et al., 2023).

## 11 Regulatory considerations

The regulations for food items provide a set of criteria regarding the nutritional profile, permitted levels of food additives, food safety guidelines, and labeling requirements. These requirements compel food manufacturers to provide adequate information regarding their products, ensuring transparency within the supply chain. In this way, regulatory bodies help consumers make informed choices that align with their dietary preferences, health goals, and cultural and religious practices. These regulations inhibit deceptive food practices, ensure food safety, promote fair food trade between countries, and enforce moral and ethical food production.

Different regulatory bodies are operating in various regions of the world. Codex Alimentarius Commission (CAC) is the regulatory authority that sets the reference standard for the majority of food and beverages. However, CAC has not recommended any specific standards regarding the nutritional profile and labeling of plant-based milk. According to the USDA, plant-based, non-dairy beverages must comply with local, state, and federal requirements regarding the preparation, packaging, labeling, distribution, and sale of these products. If plant-based beverages contain allergens, an allergen statement must be provided on the package. Unless otherwise specified, plant-based beverages must have a shelf-life of six months from their manufacturing date, and those beverages that are required to be refrigerated must have a shelf-life of three months from the manufacturing date if stored at 7 °C. The USDA Food Safety and Inspection Service (FSIS) can perform analytical testing on random samples. The plant-based, non-dairy beverages must be tested using methods approved by the Association of Official Agricultural Chemists (AOAC), and the results must conform to the microbiological and analytical requirements given in Table 4 (Adamczyk et al., 2022).

**Table 4:** Tests and Methods to Check Microbiological and Analytical Requirements.

Test	Method	Requirement
Sodium	AOAC 963.09, 985.35, 2011.14 or 2015.06	Must not exceed the limit specified by the purchaser in the solicitation, contract, or purchase order
Fat	AOAC 932.06, 983.23, 996.06 or 2008.06	Must not exceed the limit specified by the purchaser in the solicitation, contract, or purchase order
Aflatoxin	AOAC 991.31 or 998.03	Less than 20 ppb
*Salmonella*	AOAC 967.26, 967.28, 996.08, 2003.09, 2004.03, 2011.03, 2011.17, 2013.09 or BAM Ch. 5	Must be negative
*Escherichia coli (E. coli)*	AOAC 991.14, 2011.17, or BAM Ch. 4	Less than 3 Colony Forming Units (CFU) per gram or Most Probable Number (MPN) per gз
*Listeria monocytogenes (L. monocytogenes)*	AOAC 2003.12, 2013.11, 2016.08, or BAM Ch. 10	Must be negative

Plant-based milk products are non-standardized food items, as no standards have been set by the FDA for these products. Plant-based milk products must be labeled with their common name or a statement that reveals the identity of these food items. Similarly, the nutritional composition of plant-based milk depends on the chosen plant, processing methods, and added ingredients. In February 2023, the FDA released and distributed a draft guidance regarding the nutritional declaration, consumer perception, and dietary differences of plant-based milk as compared to animal milk. In 1973, the FDA published the following definition of milk:

“The lacteal secretion, practically free from colostrum, obtained by the complete milking of one or more healthy cows.”

Any product labeled as ‘milk’ must conform to this definition and should have the required standards and nutritional profile that is similar to conventional milk. As plant-based milk does not meet the criteria set by this definition, PBMAs must not be packaged or sold as ‘milk’ under the Food, Drug, and Cosmetic (FD&C) Act (Program, 2025a).

In 2017, the European Court of Justice defined the term ‘milk’ as mammary secretion obtained from one or more milkings without adding or extracting any substance from the said secretion (Scholz-Ahrens, Ahrens, & Barth, 2020). Concerning this definition, PBMAs cannot be labeled as ‘milk’.

### 11.1 A need for regulatory reforms

It has been noticed that neither the FDA, EFSA, nor Codex Alimentarius has recommended any specific regulations regarding the nutrient labeling of plant-based milk. According to the FDA, consumer reports (Program, 2025b) have revealed that consumers who purchase plant-based milk understand that PBMAs are different from milk. However, 53 % of people admitted that they believe that plant-based products labeled as ‘milk’ and milk have the same nutritional content. Similarly, 48 % of consumers believe that plant-based milk has more essential nutrients than cow's milk. 58 % of consumers who purchase plant-based milk believe it is healthier than cow's milk (Boaitey & Minegishi, 2020).

## 12 Future research

The survey mentioned above indicates that plant-based consumers lack a basic understanding of the ingredients, nutritional composition, and processing methods of various PBMAs. Regulatory authorities need to have strict policies regarding the nutrients and packaging of plant-based milk to ensure that consumers receive accurate information so they can confidently navigate the marketplace and incorporate diverse food options into their diet.

PBMAs are an expanding market, and people who prefer to utilize these beverages must be provided with an alternative that is nutritious. Even though PBMAs have a variety of essential amino acids, minerals, and vitamins, their nutritional composition is not comparable to that of dairy milk. Several treatments, including fortification and enrichment, can enhance the nutritional properties of PBMAs. Additionally, the effects of processing conditions and storage time on the bioavailability of the nutrients present in PBMAs should also be investigated.

The retail price of commercially available PBMAs varies widely. On average, PBMAs are 2 to 5 times more expensive than cow's milk (Scholz-Ahrens et al., 2020). Such processing technologies must be investigated that can reduce the overall cost of various PBMAs.

## 13 Conclusion

This review study aims to provide an in-depth appraisal of PBMAs. It covers nutritional, sensorial properties, processing, trends in the international market, allergenicity, health implications, consumer choices, and labeling regulations. With the increasing interest of consumers in health and the environment, a comparison of PBMAs with cow milk on different factors has been done to distinguish between the two kinds of milk. The need for PBMAs has increased. Keeping in mind that they are milk substitutes, not a replacement. Supplementation and fortification are done to overcome this issue for the masses. They are rich sources of proteins and contain certain amounts of PUFAs and MUFAs. PBMAs contain essential vitamins and minerals, but they lack vitamin D, vitamin B12, and some other minerals. The nutritional content is also determined by the processing it goes through. PBMAs are a rich source of antioxidants that help in protecting the cardiovascular system. Many health benefits include the presence of bioactive compounds and phytochemicals, free of cholesterol and lactose. With the health benefits, they also pose threats concerning allergens. Many plant milks pose threats of allergies, such as peas, soy, buckwheat, peanut, almond, and coconut, etc. It mediates IgE reactions in the body. One of the limitations of this study is that there is a vast range of plant sources that are used to make plant milk; in this study majority of them are covered, but there is a high chance that some might be left. Information regarding all the plant milk sources should be gathered in one place. The world international market for these products is becoming a huge business, which is expected to reach a value of 26 billion USD in the next five years. Consumers are seeking alternatives to milk, keeping in mind its impacts on health and the environment. Its demand has increased manyfold in the past years. Production of cow milk has shown a negative impact on the environment in terms of greenhouse gas emissions, water, and land resources, etc. PBMAs also contribute to all these factors, but in smaller amounts. There is a lack of regulations for the nutritional fact labels, packaging, storage requirements, etc., regarding plant-based milk, which causes variation. To overcome this issue, regulatory bodies should work on the standardization of various nutritional milk fact labels and all other important parameters to ensure their quality and safety.

## CRediT authorship contribution statement

**Noor Asif:** Writing – original draft, Software, Resources, Investigation, Data curation, Conceptualization. **Oneeza Anwar:** Writing – original draft, Resources, Project administration, Investigation, Formal analysis, Data curation. **Sabika Arif:** Writing – review & editing, Software, Investigation, Formal analysis, Data curation, Conceptualization. **Zahra Anwar:** Writing – review & editing, Validation, Software, Resources, Methodology, Investigation, Formal analysis. **Iahtisham-Ul-Haq:** Writing – review & editing, Writing – original draft, Supervision, Resources, Project administration, Investigation, Formal analysis, Data curation. **Sezai Ercisli:** Writing – review & editing, Software, Methodology, Funding acquisition, Formal analysis, Data curation. **Robert Mugabi:** Writing – review & editing, Resources, Formal analysis, Data curation, Conceptualization. **Gulzar Ahmad Nayik:** Writing – review & editing, Supervision, Software, Methodology, Investigation, Data curation.

## Declaration of competing interest

The authors declare that they have no known competing financial interests or personal relationships that could have appeared to influence the work reported in this paper.

## Data Availability

No data was used for the research described in the article.
